# Selective alterations of neurons and circuits related to early memory loss in Alzheimer’s disease

**DOI:** 10.3389/fnana.2014.00038

**Published:** 2014-05-27

**Authors:** Maria Llorens-Martín, Lidia Blazquez-Llorca, Ruth Benavides-Piccione, Alberto Rabano, Felix Hernandez, Jesus Avila, Javier DeFelipe

**Affiliations:** ^1^Consejo Superior de Investigaciones Cientificas–Universidad Autónoma de Madrid, Centro de Biología Molecular Severo Ochoa, Universidad Autónoma de MadridMadrid, Spain; ^2^Laboratorio Cajal de Circuitos Corticales, Centro de Tecnología Biomédica, Universidad Politécnica de MadridMadrid, Spain; ^3^Instituto Cajal, Consejo Superior de Investigaciones CientificasMadrid, Spain; ^4^Centro de Investigación en Red sobre Enfermedades NeurodegenerativasMadrid, Spain; ^5^Departamento de Neuropatología y Banco de Tejidos, Fundación CIEN, Instituto de Salud Carlos IIIMadrid, Spain

**Keywords:** cerebral cortex, pyramidal cells, granule cells, neurogenesis, dendritic spines, hippocampal connections, spread of tau, amyloid beta pathology

## Abstract

A progressive loss of episodic memory is a well-known clinical symptom that characterizes Alzheimer’s disease (AD). The beginning of this loss of memory has been associated with the very early, pathological accumulation of tau and neuronal degeneration observed in the entorhinal cortex (EC). Tau-related pathology is thought to then spread progressively to the hippocampal formation and other brain areas as the disease progresses. The major cortical afferent source of the hippocampus and dentate gyrus is the EC through the perforant pathway. At least two main circuits participate in the connection between EC and the hippocampus; one originating in layer II and the other in layer III of the EC giving rise to the classical trisynaptic (ECII → dentate gyrus → CA3 → CA1) and monosynaptic (ECIII → CA1) circuits. Thus, the study of the early pathological changes in these circuits is of great interest. In this review, we will discuss mainly the alterations of the granule cell neurons of the dentate gyrus and the atrophy of CA1 pyramidal neurons that occur in AD in relation to the possible differential alterations of these two main circuits.

## A NOTE ON THE GENERAL ANATOMICAL CHARACTERISTICS OF THE HIPPOCAMPAL FORMATION

The term hippocampus is commonly used in its broad sense, to include the dentate gyrus (DG) and the hippocampus proper, or Ammon’s horn, while the DG is sometimes considered a separate structure of the hippocampus. In general, the hippocampal formation includes the DG, the hippocampus proper and the subicular complex (which includes the subiculum, presubiculum, and parasubiculum). However, other authors also consider the entorhinal cortex (EC) as part of the hippocampal formation (Insausti and Amaral, 2004). Following the nomenclature of Lorente de Nó (1934), the hippocampus proper is subdivided into the CA1, CA2, CA3, and CA4 fields. The Ammon’s horn neurons enclosed within the concavity of the granule cell layer constitutes the CA4 field. However, as pointed out by Insausti and Amaral (2004), the term CA4 is confusing since it is often applied either to the polymorphic layer (or hilus) of the DG (see below) or to the inserted portion of the pyramidal cell layer of CA3. Indeed, for practical topographic reasons, the term CA4 is often used to refer to “the region within the C-shaped DG” or “C4-DG” in studies of human magnetic resonance images. Furthermore, there is a lack of clear cytoarchitectonic characteristics or connections that distinguish pyramidal CA3 neurons from those of the CA4 and thus it seems to be more appropriate to refer to these fields simply as the CA3 (Insausti and Amaral, 2004). For the purposes of the present work, the hippocampal formation will refer to the DG, the hippocampus proper (subdivided into three fields: CA1, CA2, and CA3) and the subicular complex.

In the hippocampal formation, there are two main classes of neurons (reviewed in Freund and Buzsaki, 1996; Amaral and Lavenex, 2007; Bezaire and Soltesz, 2013): the principal (or projection) neurons that represent the majority of neurons, and short-axon cells (or interneurons). Principal neurons are excitatory (glutamatergic), have spiny dendrites and include: the granule cells of the DG; the mossy cells of the dentate hilus; pyramidal neurons of Ammon’s horn; and the pyramidal cells of the subicular complex. Interneurons are inhibitory (GABAergic), have varied morphological and neurochemical features and are found in all regions and layers of the hippocampal formation. The vast majority of neurons in the DG are granule cells, whereas in the Ammon’s horn and subiculum the most common neuron type is the pyramidal cell. Accordingly, the cell layers in the hippocampus are referred to as the granule cell layer (in the case of the DG) or the pyramidal cell layer (in the rest of the fields). The layers above and below the granule cell layer are called the molecular layer and the polymorphic cell layer (or hilus), respectively. The *stratum radiatum* (formed mainly by the apical dendrites of pyramidal neurons) and the *stratum lacunosum-moleculare* (adjacent to the hippocampal fissure) lie above the pyramidal cell layer in the CA1 - CA3 fields. In addition, in the rat and monkey the *stratum lucidum* can be distinguished. This stratum is formed mainly of mossy fiber bundles and lies just above the CA3 pyramidal cell layer. In humans, this layer is hard to identify because numerous mossy fibers run through the pyramidal cell layer. The layers below the pyramidal cell layer of CA1 - CA3 are the *stratum oriens* and the alveus, which are comprised mainly of pyramidal cell axons. The layers above and below the pyramidal cell layer of the subiculum are the molecular layer and the angular bundle, respectively. Furthermore, some authors subdivide the subiculum into two cellular sublayers: the external or superficial layer (close to the *stratum radiatum*); and the internal or deep layer (facing the *stratum oriens*). According to Insausti and Amaral (2004), the presubiculum is characterized by a single, superficial layer (layer II) that is made up of small, densely packed neurons situated in a narrow lamina at the posterior levels, whereas they form large spherical islands at the rostral levels. The parasubiculum is also made up of a single cellular layer (layer II) that contains larger and less densely packed neurons than the presubiculum.

**FIGURE 1:**
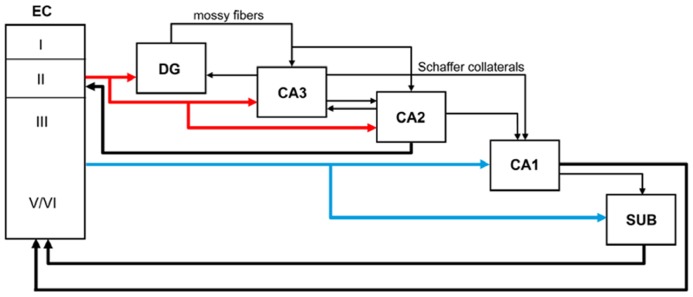
**Schematic diagram showing the main perforant pathway projections to the hippocampus and the main connections between the dentate gyrus (DG), CA fields, subicular complex (SUB), and entorhinal cortex (EC)**.

The major cortical input to the hippocampus and dentate gyrus is the EC through the so-called perforant pathway. This pathway arises mainly from layers II and III (**Figure 1**). Neurons in layer II project to the DG and CA3, whereas layer III cells project to CA1 and the subiculum. Furthermore, in the monkey it has been shown that neurons in layer V also project to the CA1/subiculum, and neurons in layer VI project to DG and CA3 - although both of these types of projections occur to a lesser extent than those in the superficial layers (Witter et al., 2000; Insausti and Amaral, 2004). The perforant pathway can be subdivided into two projection systems named the lateral and medial perforant pathway; one originating in the lateral EC (LEC) and the other in the medial EC (MEC). Cells in layer II of the LEC project to the outer third of the molecular layer of the DG and outer half of the *stratum lacunosum-moleculare* of CA3, whereas layer II cells of the MEC establish connections with the middle third of the molecular layer of DG and inner half of the *stratum lacunosum-moleculare* of CA3. Cells in layer III of the LEC project to the distal part of CA1 and the proximal part of the subiculum, whereas those layer III axons originating in the MEC connect with the proximal part of CA1 and the distal part of the subiculum. These EC layer III axons form synapses on the most distal portion of the apical dendrites (*stratum lacunosum-moleculare*) of CA1 pyramidal cells. The axons of the granule cells of the DG are called mossy fibers and they form synapses with the polymorphic neurons of the DG and CA3 neurons. In turn, CA3 pyramidal neurons represent a major afferent system to the CA1 through the so-called Schaffer collaterals, which form synapses on the apical dendrites (*stratum radiatum*) and basal dendrites (*stratum oriens*) of CA1 pyramidal cells (Amaral and Witter, 1989). These two pathways (ECIII → CA1 and ECII → dentate gyrus → CA3 → CA1) differentially influence activity in CA1. However it has been shown that both projection systems convergence on single CA1 pyramidal cells (Kajiwara et al., 2008). CA1 pyramidal cells mostly innervate the subiculum and this field establishes connections with the pre- and parasubiculum. The neurons in the subiculum, as well as those in both the pre- and parasubiculum, project to the EC where they terminate in layers IV to VI (Witter et al., 2000; Insausti and Amaral, 2004; Witter, 2007). Recently, the CA2 field, which is commonly not included in the hippocampal circuitry, has been shown to have an important role in hippocampal circuitry and memory function in the mouse (Chevaleyre and Siegelbaum, 2010; Jones and McHugh, 2011; Cui et al., 2013; Rowland et al., 2013; Kohara et al., 2014). According to these studies DG cells also project to CA2, which in turn connects mainly with CA1. The pyramidal cell axons of CA2 terminate in *stratum oriens* and *stratum radiatum* of CA1. Furthermore, CA3 and CA2 are mutually connected and these connections are dominated by inhibition (Kohara et al., 2014). Finally, CA2 receives input from layer II of the EC and CA2 also projects back to this layer. Thus, CA2 is involved in a trisynaptic circuit that is different from the classic trisynaptic hippocampal circuitry (**Figure 1**).

Finally, it should be noted that the pattern of connections described above is based on studies performed mainly in rats, cats, monkeys and - most recently - in mice. Although similar connectivity appears to exist in all species studied, there are also variations. Thus, at present we can only make the assumption that the pattern of connections of the human hippocampal formation is basically similar to that described in experimental animals, but we cannot rule out the possibility of marked differences.

## THE PROGRESSION OF AD BASED ON THE HIPPOCAMPAL CIRCUITRY: THE LOSS OF MEMORY

Scoville and Milner (1957) made a seminal observation; that a bilateral medial temporal resection in an epileptic patient correlates with the loss of declarative memory. A deeper understanding of this type of memory came later with the studies of Cohen and Squire (1980). The components of the removed medial temporal lobe included the CA fields of the hippocampus, the DG, the subiculum and the surrounding cortex (entorhinal, perirhinal, and parahippocampal cortices). In attempting to determine the minimal anatomical component involved in memory impairment, it is worth noting that observations of other patients with memory problems, led to the description of loss of memory in response to viral encephalitis (Damasio et al., 1985), posterior artery occlusion (Benson et al., 1974), or hypoxic ischemia (Volpe and Hirst, 1983), affecting CA1. Posterior studies showed that lesions centering on CA1 were sufficient for memory impairment (Zola-Morgan et al., 1986).

A main feature occurring in Alzheimer disease is the loss of declarative memory at early stages of the disease and this impairment was correlated with damage in the upper layers of EC (loss of neurons in layer II; Van Hoesen et al., 1991; Gomez-Isla et al., 1996). Indeed, it has been described that a specific region of EC is damaged at early stages of AD, and that the efferent connectivity emerging from this area is impaired (Van Hoesen et al., 1991; Gomez-Isla et al., 1996), with reports of the LEC in particular being affected in preclinical disease (Khan et al., 2014).

The presence of tau pathology (pre-tangles and tangles) in the LEC/transentorhinal area has been reported as representing the initial stage of Alzheimer’s neurofibrillary degeneration (Braak stage I) in the medial temporal lobe (Braak and Braak, 1991; Braak et al., 2006; Lace et al., 2009). However, it is important to address the question of whether these pre-tangles and tangles really are indicative of incipient AD or whether they are independent, aging-related changes. Longitudinal studies on aging and dementia have shown that in postmortem brain series the majority of cognitively intact aged subjects display some degree of tau-related pathology with low Braak stages (Sonnen et al., 2011). These changes have been envisioned by some authors as representing preclinical AD, which may follow predictable successive neuropathological stages involving both neurofibrillary and beta-amyloid pathologies (Jicha et al., 2012). Within the current theoretical framework for AD pathogenesis (Hyman, 2011), there is still an ongoing debate between authors that consider low Braak stages as a common trait of brain aging (von Gunten et al., 2010) and those that emphasize their pathological nature (Ferrer, 2012). Current evidence suggests that while it is true that the pathology of AD is associated with advanced age, there is still a low proportion of aged non-AD subjects that develop no neurofibrillary pathology whatsoever (Sonnen et al., 2011; Jicha et al., 2012) suggesting that such pathology is not simply an inevitable consequence of age. Additionally, the oldest old do not show a lineal correlation between age and neurofibrillary pathology, and genetic traits associated with accelerated aging do not entail a higher risk for AD (Nelson et al., 2011). Hence, the most widely held view is that, although highly prevalent among aged subjects in general, low Braak stages of neurofibrillary pathology do correspond to the initial phases of AD. Furthermore, several researchers have hypothesized the existence of a variety of functional compensatory mechanisms that may play a role in the development of dementia by cognitively normal people with low Braak stages. According to Lazarczyk et al. (2012), the compensatory mechanisms could be considered passive or active. The former would be related to cognitive reserve and would only delay the conversion to dementia, whereas the active compensatory mechanisms would be continuously acting in the brain, stopping the progression of the disease and, therefore, preventing the conversion to dementia. The study of these compensatory mechanisms is clearly important, given their potential as new targets for therapeutic interventions for AD.

Since EC gives rise to the major cortical afferent source of the hippocampus and DG through separate pathways, the study of the early pathological changes in the target fields of the perforant pathway is of great interest. The classic trisynaptic circuit EC layer II → DG → CA3 → CA1 seems to be related to the acquisition of new memories, whereas the interaction between EC and CA1 neurons (monosynaptic pathway) is thought to contribute to the strength of previously established memories (Cohen and Squire, 1980). In this review, we shall comment on some main regions of the hippocampal formation that, on the basis of their connections with the EC, might be affected in AD patients.

### THE SPREAD OF TAU AND ABETA PATHOLOGY

**FIGURE 2:**
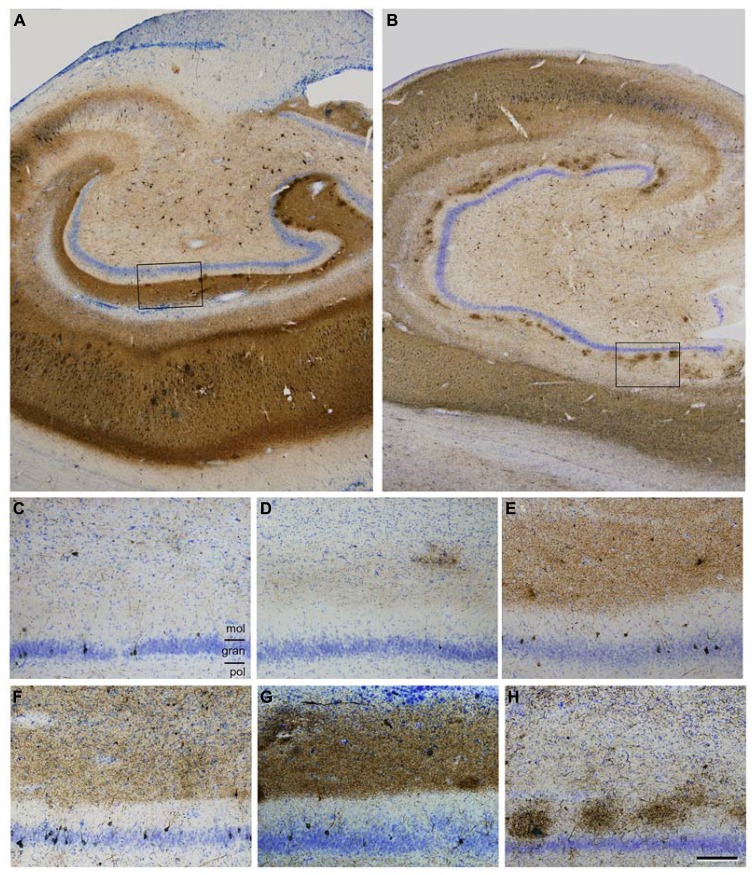
**(A,B)**, Low power photomicrographs of anti-PHF-tauAT8 stained sections counterstained with the Nissl technique showing different fields of the hippocampal formation of patients P9 **(A)** and P10 **(B)**. Boxed areas in **(A,B)** correspond to **(G,H)**, respectively. **(C-H)**, High magnification of the dentate gyrus of patients P6, P5, P11, P7, P9 and P10, respectively, to illustrate different patterns of PHF-tau_AT8_ immunostaining. Scale bar **(H)**: 700 μm in **(A,B)**; 130 μm in **(C-H)**. gran = granular layer; mol = molecular layer; pol = polymorphic layer. Taken from Blazquez-Llorca et al. (2011).

**FIGURE 3:**
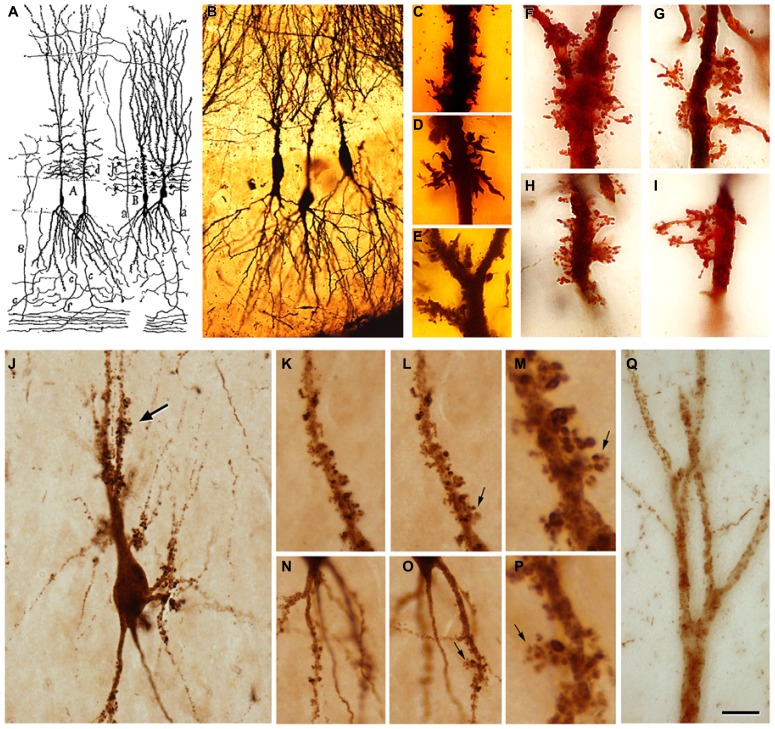
**(A)** Drawing by Cajal showing pyramidal cells with thorny excrescences in the CA3. **(B)**, Cajal’s original histological preparations from the rabbit CA3 stained by the Golgi method. **(C-I)**, Examples of thorny excrescences on CA3 pyramidal neurons. **(C-E)**, Dendrites from a newborn child’s CA3 pyramidal neurons stained by the Golgi method; **(F-I)**, Dendrites of rabbit CA3 pyramidal neurons stained by the Kenyon’s variant of the Golgi method (preparations housed at the Cajal Institute). Thorny excrescences were named and discovered by Cajal in the apical dendrites of rabbit CA3 pyramidal neurons (Estructura del asta de Ammon y fascia dentata. *Anales de la Sociedad Española de Historia Natural*, tomo XXII, 53–114, 1983). He correctly proposed that these large and often branched structures served as points of contact with the mossy fibers from the dentate gyrus. The presence of excrescences was later confirmed in a variety of species, including humans, both in the apical and basal dendrites and in the hilar mossy cells. These complex dendritic spines represent a major target of the axon terminals (mossy fibers) of hippocampal dentate granule cells. The thin unmyelinated mossy fibers form numerous large, *en passant* swellings and terminal expansions (giant mossy fiber boutons), which establish numerous synapses with the thorny excrescences. **(J)**, PHF-tau_AT8_-ir CA3 pyramidal neuron from patient P4 exhibiting a cluster of thorny excrescences (arrow). **(K,L**,**N,O)**, Two focal planes showing thorny excrescences on CA3 PHF-tau_AT8_-ir cells from the same patient. **(M,P)**, Higher magnification of the thorny excrescences (arrows) in **(L,O)**, respectively. **(Q)**, Apical dendritic shaft of a CA2 PHF-tau_AT8_-ir pyramidal cell with no labeled dendritic spines. Scale bar **(Q)**: 55 μm in **(B)** 11 μm in **(C-I)**; 25 μm in **(J)**; 20 μm in **(K,L,N,O)**; 4 μm in **(M,P)**; 10 μm in **(Q)**. Taken from Blazquez-Llorca et al. (2011).

There are numerous articles suggesting that the progression of AD is based on anatomical connections and can propagate as a prion-like disease (e.g., Lace et al., 2009; Jucker and Walker, 2013). In these studies, the degree of tau and amyloid beta (Aβ) pathology in the main target regions of the EC has been associated with the degree and course of cognitive impairment in these patients, with the association being stronger in the case of tau (see also Nelson et al., 2012). This overall observation is based on the fact that the tau and Aβ pathology found in any region of the hippocampal formation of AD patients is rather variable (low, moderate, strong, or very strong) and intuitively seems to indicate a progressive alteration (Braak and Braak, 1991). For example, tau pathology, as revealed with tau monoclonal antibodies AT8 or PHF-1 (PHF-tau_AT8_ and PHF-tau_PHF-1_), shows different patterns of immunostaining in different AD patients that could be interpreted as steps of the progression of the disease. In **Figures 2C–H** show sections of the molecular layer of the DG, immunostained for PHF-tau_AT8_, from different patients - with the panels (C–G) ordered in such a way that each one is representative of what would be a progressive step. Nevertheless, if tau deposition progresses in a hierarchical manner, and we assume that all neurons that are connected with the initially damaged neuron are “infected,” the spread of abnormal tau should be present in the main target regions of the perforant pathway, that is, the DG, CA3, CA1, and subiculum. However, our results suggest that at least in some patients, the DG does not seem to be involved in this propagation as relatively few granule cells are phosphotau positive despite the intense PHF-tau_AT8_ labeling of the outer molecular layer that occurs in these patients (e.g., see **Figure 2A**). In this respect, in a recent study, we found a conspicuous abnormal phosphorylation of tau in the typical thorny excrescences of hippocampal CA3 neurons in a pre-tangle state (pattern I, see below), which was independent of the stage of AD pathology and of the intensity of the phosphotau immunostaining in the DG (Blazquez-Llorca et al., 2011; **Figure 3**). Since thorny excrescences represent a major synaptic target of granule cell axons (mossy fibers), we suggested that such aberrant phosphorylation may play an essential role in the memory impairment typical of AD patients, independent of the presence or intensity of phosphotau expression of EC axons innervating their dendritic arbor.

Another issue is the rather variable degree of atrophy of the CA1 between patients (compare the thickness of CA1 shown in **Figure 2A** with that illustrated in **Figure 2B**). This atrophy indicates neuronal loss and/or atrophy of the pyramidal dendritic arbors in the CA1 pyramidal cell layer. Therefore, in these patients, it seems reasonable to suggest that the functional consequences of the alterations of anatomical pathways and the presumptive spread of the pathology to the target regions where the CA1 pyramidal cells project would be different from those patients with less atrophy.

**FIGURE 4:**
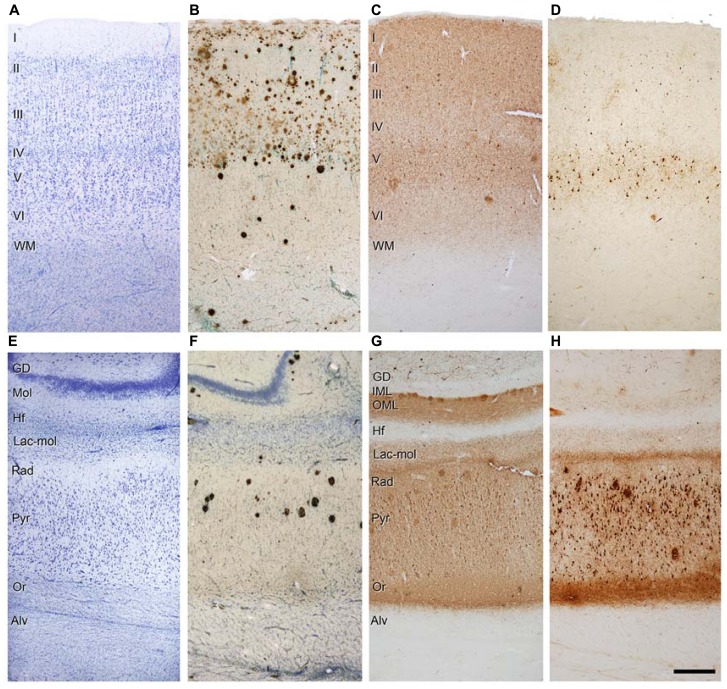
**Photomicrographs of sections from the parahippocampal cortex (A-D) and CA1 region (E-H) of patient P9 stained for Nissl (A,E), Aβ/Nissl (B,F), PHF-tau_**AT8**_**(C,G)**, and PHF-tau_**PHF-1**_ (D,H)**. Alv, alveus; DG, dentate gyrus; Hf, hippocampal fissure; IML, inner molecular layer; Lac-mol, stratum lacunosum-moleculare; Mol, molecular layer; OML, outer molecular layer; Or, stratum oriens; Pyr, stratum pyramidale; Rad, stratum radiatum; WM, white matter; Scale bar **(H)**: 250 μm in **(A-H)**. Taken from Merino-Serrais et al. (2013).

Similarly, Aβ pathology is also variable and affects different regions and layers (**Figure 4**). For example, in the case shown in **Figure 4B**, numerous Aβ plaques are found in the parahippocampal region in layers I – IV, whereas in layers V – VI there are very few Aβ plaques, and in the pyramidal cell layer of CA1 they are found practically only in its superficial aspect (**Figure 4F**). Thus, the connectivity of these upper layer neurons must be more profoundly altered than in the lower layer neurons. In addition, immunostaining for PHF-tau_AT8_ and PHF-tau_PHF-1_indicates that different circuit might be altered depending on the sites of the phosphorylation of tau. In **Figures 4C,G**, a moderate but widespread pathology with PHF-tau_AT8_ is shown in the parahippocampal cortex and CA1 region, respectively, whereas with PHF-tau_PHF-1_, layer V of the parahippocampal cortex is heavily affected but none of the other layers are significantly affected (**Figure 4D**). CA1 is much more affected than the parahippocampal region with a heavy PHF-tau_PHF-1_ staining of the pyramidal cell layer, the superficial part of the *stratum lacunosum-moleculare* and *stratum oriens* of CA1 (**Figure 4H**). Interestingly, as shown in **Figures 4G,H**, the outer molecular layer of the DG shows a heavy PHF-tau_AT8_ staining but not in the case of PHF-tau_PHF-1_. Thus, it is clear that different pathways are affected selectively depending on the tau phosphorylation sites. Whether the hypothetical spread of tau pathology is through the anatomical connections and whether this depends on the type of tau phosphorylation remains to be elucidated. With regards to this, it should be noted that previous results suggest that tau, and not phospho-tau, is the main factor triggering the spread of tau pathology (Diaz-Hernandez et al., 2010).

Finally, it is logical to hypothesize that if progression of tau pathology is based on anatomical connections, then this must occur through the axons of the glutamatergic projections cells, i.e., the granule cells and pyramidal cells. However, the question remains as to what happens to the GABergic interneurons that are innervating these projection cells. It is well established that parvalbumin-ir neurons represent a large subpopulation of cortical GABAergic interneurons that includes basket and chandelier cells (e.g., DeFelipe, 1993; Freund and Buzsaki, 1996). These cells provide the main GABAergic innervation to the soma, proximal dendrites and axon initial segments of pyramidal cells. However, in a previous study on the pericellular innervation of neurons expressing abnormally hyperphosphorylated tau in AD patients, we found that the vast majority of parvalbumin-ir cells did not contain PHF-tau (Blazquez-Llorca et al., 2010). To quantify this observation, we counted the number of PV-ir neurons that were also PHF-tau-ir and we observed that of the 3943 PV-ir neurons analyzed in the hippocampal formation and EC of AD patients only two cells were PHF-tau-ir. This is in line with other studies suggesting that GABAergic circuits in the cerebral cortex of AD patients are relatively well preserved when compared to circuits that use other neurotransmitters. For example, the subpopulation of parvalbumin-ir cells apparently remains unaltered in the prefrontal and inferior temporal cortex (Hof et al., 1991) and in the temporal cortex (Ferrer et al., 1991). However, other studies indicated possible alterations to the parvalbumin-ir basket and chandelier cells in the cerebral cortex of AD patients, but as discussed previously (Blazquez-Llorca et al., 2010), the discrepancy between different laboratories regarding the loss or preservation of GABAergic neurons may be explained by differences in tissue processing, the methods of analysis (quantitative versus qualitative studies), and/or the postmortem delay, together with the clinical variability between patients and control tissue.

Thus, caution is needed when interpreting pathological findings as indicating a connection alteration. Nevertheless, it may be that particular excitatory circuits are affected, and cytoskeletal changes may be related to disconnection of excitatory circuits.

### ALTERATION OF NEURON MORPHOLOGY

It is important to summarize the changes in the morphology of the dendritic arbor and spines of granule neurons of the DG (Llorens-Martin et al., 2013) and changes in the number and volume of spines of dendrites from CA1 neurons in patients with AD (Merino-Serrais et al., 2013). These changes are indicative of alterations in the synaptic input of these neurons and this is further discussed below.

#### Granule cells

The morphology of mature granule neurons present in the DG has been extensively characterized (Zhao et al., 2008) and it has been described that a specific Y-like morphology was required for the proper function of these neurons (Zhao et al., 2008; Llorens-Martin et al., 2013; Khan et al., 2014).

**FIGURE 5:**
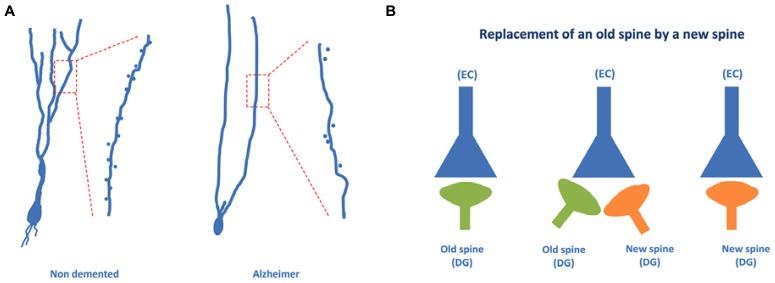
**Granule neurons of the dentate gyrus form functional synapses.** In **(A)**, the typical “Y” shape morphology characteristic of granule neurons can be observed in the case of non-demented control cases, whereas an altered, “V” shape morphology has been described in the case of AD patients. **(B)** Illustrates the steps involved in the synaptic replacement of an old spine with a new one. It should be noted that granule neurons of AD patients show a drastically reduced density of dendritic spines.

Recently, using the Golgi method we have found that a high proportion of granule neurons exhibit a V-like shape morphology (that is, more than one primary apical dendrite emerging directly from the soma) in AD patients, whereas a high proportion of granule neurons showing a Y-like shape was found in the case of non-demented control cases (Llorens-Martin et al., 2013; see **Figure 5A**). In addition, a lower density of dendritic spines was found in granule neurons of AD patients as compared to non-demented controls.

Dendritic spines of granule cells undergo a continuous and dynamic process which seems to be related to the formation of new synapses that are needed for the integration of adult-born granule neurons into the glutamatergic network (Toni et al., 2008; Toni and Sultan, 2011) - a crucial step for the acquisition of new hippocampal related memories (Zhao et al., 2008).

It has been suggested that the formation of a new synapse in a newborn neuron may involve the appearance of a new dendritic spine and that this new dendritic spine can replace an older dendritic spine of an older neuron (Toni and Sultan, 2011). This synaptic reshaping may result in the replacement of an old connection with a new connection (**Figure 5B**). The data regarding AD patient granule neurons may suggest that this connection replacement is not taking place properly in the DG and that, consequently, the proportion of old connections may be high. The result may be an impairment of the formation of new memories, which involves a process known as pattern separation (Rolls, 2013). In this process, occurring at the DG, it appears that a new memory is differentiated from older memories involving similar but not identical facts. Thus, regarding the pathway EC → DG → CA3 → CA1, it can be proposed that in AD patients the new connections between EC neurons and DG newborn neurons are likely impaired.

#### CA1 pyramidal neurons

Atrophy of the hippocampus is a well-known pathological feature of AD which intuitively indicates that the dendritic arbor of pyramidal cells is reduced concomitantly. Importantly, there is a clear atrophy of the CA1-*stratum radiatum/stratum lacunosum-moleculare* in patients with mild AD (Kerchner et al., 2012), which suggests an early alteration in AD. The question remains as to what the influence of the presence of phosphotau is in the microanatomy of pyramidal cells. To address this question, we have recently used intracellular injections of Lucifer yellow in fixed tissue from AD patients, to analyze dendritic spines that were completely reconstructed in three dimensions along the length of the basal dendrites of pyramidal neurons in the parahippocampal cortex and CA1 at the same rostrocaudal level (Merino-Serrais et al., 2013). Following intracellular injection, sections were immunostained for anti-Lucifer Yellow and with antibodies AT8 and PHF-1. Thus, we were able to examine possible changes in the microanatomy of dendrites from pyramidal neurons with tau pathology. We observed that at the early stages of a neurofibrillar pathology [as defined by the presence of diffuse phosphotau in a putative pre-tangle state (pattern I)], pyramidal neurons do not show alterations. However, once tau aggregates of intraneuronal neurofibrillary tangles had formed (patterns IIa and IIb), representing more advanced neurofibrillar alterations, significant reductions in the diameter of dendrites and in the number, length and volume of spines were observed. Furthermore, we proposed that the severity of these changes was likely to be progressive, from the intermediate/advanced stages (pattern IIa) to the extreme stage (pattern IIb) of the neurofibrillar pathology leading to loss of dendritic spines and dendritic atrophy (see below). It is well established that the loss of dendritic spines indicates a synaptic disconnection, which clearly must have an important consequence in the cognitive attributes of the AD patients. Thus, we concluded that the presence of phosphotau in neurons does not necessarily mean that they suffer severe and irreversible effects, but rather the characteristic cognitive impairment in AD is likely to depend on the relative number of neurons that have well developed tangles (Merino-Serrais et al., 2013).

## DIFFERENTIAL ALTERATIONS OF MONOSYNAPTIC AND TRISYNAPTIC CIRCUITS

**FIGURE 6:**
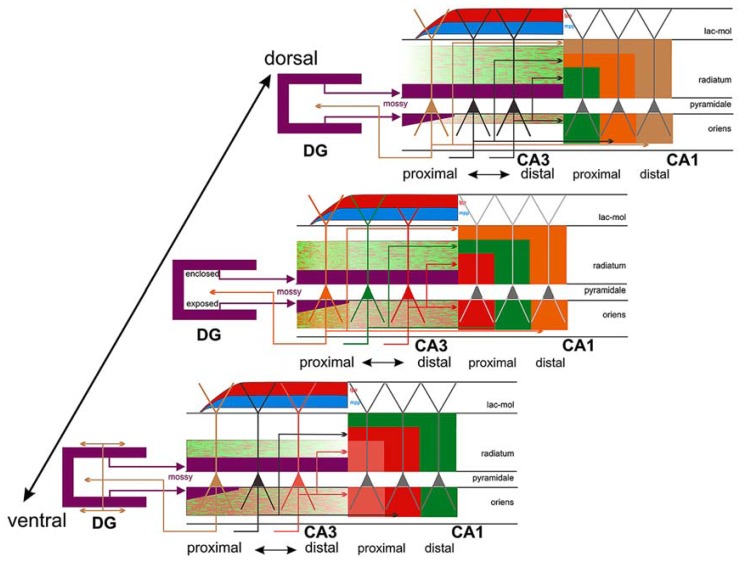
**Connections of CA3: three different levels (panels) along the dorsoventral (septotemporal) axis of CA3 are shown, bordered by CA1 and DG.** The central panel shows three color-coded neurons with different positions along the transverse (proximo-distal) axis of CA3. Collateral distribution is shown for both the association and Schaffer system along the transverse and longitudinal axes, using color coding related to the parent cell bodies in the central panel. Note the transverse shifts of both the association and the Schaffer system along the long axis but in the opposite direction. Also indicated is the radial shift of the association system along the longitudinal axis as well as the very limited longitudinal and transverse extent of the proximally arising association system (orange/green terminal field in *radiatum and oriens* at the level of the central panel only). This contrasts strongly with the more widespread distribution of the more distally arising components (green/red terminal fields in all three panels). Cells in proximal CA3, receiving less perforant path input (indicated by red and blue, lateral [lpp] and medial [mpp], respectively) but strong infrapyramidal mossy fiber input (purple), project back to the hilus of the dentate gyrus, with an increasing component that distributes to the inner molecular layer at more ventral levels (lower panel). Not indicated is the strong longitudinal component of the mossy fiber system that distributes along ~20–25% of the long axis of distal CA3. Taken from Witter (2007).

As shown in **Figure 6**, the proximal and distal apical dendritic portions of CA1 pyramidal cells (proximal apical portion: deep part of the *stratum radiatum*; distal apical portion: superficial part of the *stratum radiatum* and *lacunosum-moleculare*) receive different inputs, which may vary along the dorsoventral axis of the hippocampus. Thus, it is important to determine which of the two circuits of the perforant pathway is affected earlier and more by the disease, or if, in contrast, both the classical trysynaptic and monosynaptic pathways are equally affected. It is also important to address whether the pyramidal cells located at different rostrocaudal levels of the human hippocampus display the same structural alterations.

**FIGURE 7:**
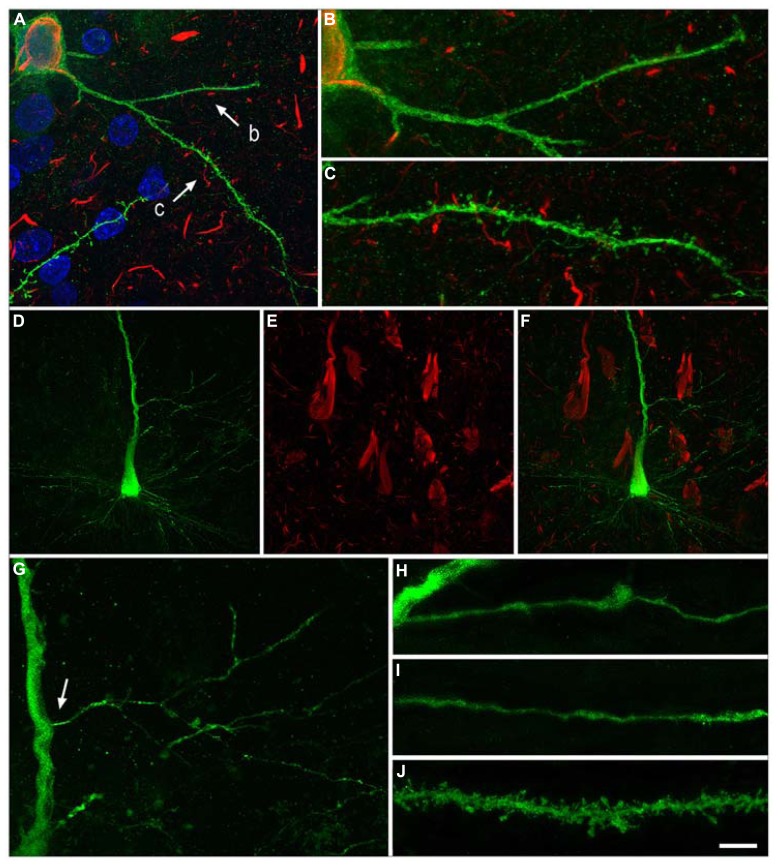
***Neurons with pattern IIb of PHF-tau immunostaining.*** Alterations of dendrites and dendritic spines in LY-injected neurons with pattern IIb PHF-tau_AT8_-ir from layer III of the PHC in patient P9 **(A-C)** and pattern IIb PHF-tau_PHF-1_-ir in the CA1 of patient P12 **(D-J)**. **(A)**, Stack of 27 confocal optical sections obtained after combining the channels acquired separately for DAPI (blue), LY (green) and PHF-tau_AT8_-ir (red), showing the cell body and proximal dendrites of the intracellular labeled neuron with pattern IIb PHF-tau_AT8_-ir. **(B,C)**, Higher magnification of **(A)**, showing the dendrites indicated as b and c, respectively. Note the low density of dendritic spines in dendrite b compared to dendrite c, suggesting that the loss of dendritic spines does not occur synchronously across the whole dendritic arbor. Also, the dendritic spines are notably smaller. **(D,E)**, Stacks of 27 confocal optical sections showing the cell body and proximal dendrites of the intracellular labeled neuron **(D)** with pattern IIb PHF-tau_PHF-1_-ir **(E)**. **(F)**, image obtained by combining **(D**,**E)**. **(G)** Higher magnification of **(D)**. **(H,I),** Stacks of 38-55 confocal optical sections from a collateral apical dendrite (arrow in **G)** of the LY-injected pyramidal neuron, showing different segments of the same dendrite **(H**, proximal; **I**, distant). **(J)**, Stack of 26 confocal optical sections from the collateral apical dendrite of an intracellularly labeled neuron that was adjacent to the LY-injected neuron shown in **(D)**, and that was not PHF-tau_PHF-1_-ir. Note the lack of dendritic spines and the thin diameter of the dendrites of the PHF-tau_PHF-1_-ir neuron **(H,I)** compared to the dendrite of the PHF-tau^-^ neuron **(J)**. Scale bar in **(J)**: 10 μm in **(A)**; 3.5 μm in **(B,C)**; 20 μm in **(D-F)**; 9 μm in **(G)**; 4.5 in **(H-J)**. Taken from Merino-Serrais et al. (2013).

**FIGURE 8:**
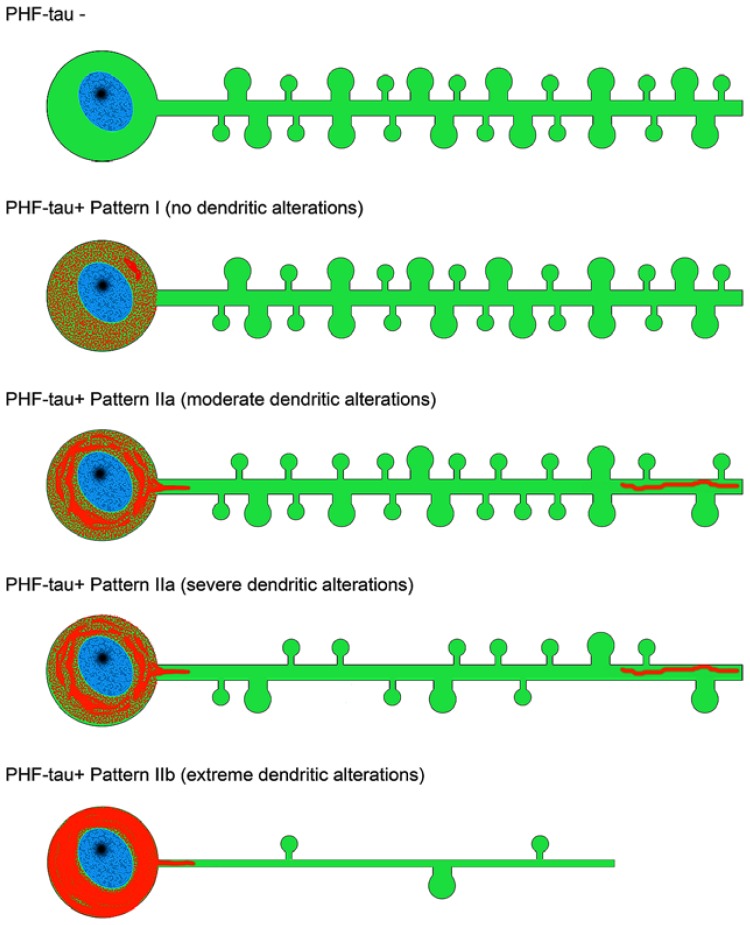
**Scheme representing the changes to dendrites of LY-injected neurons that showed different patterns of PHF-tau immunostaining.** For simplicity, dendritic spines were represented as two types: small and large. Red, phosphotau. Taken from Merino-Serrais et al. (2013).

It is well known that tau hyperphosphorylation is a pathological feature of AD (Avila et al., 2004). Furthermore, it has been proposed that tau could play a role in developing dendritic spine structure (Sapir et al., 2012), seriously threatening neuron connectivity (Ittner et al., 2010). This possibility should be confirmed because it may suggest that tau in phosphorylated form can lose the function that facilitates the development of dendritic spine structure and, thus, it may explain the observed correlation of tau phosphorylation and disappearance of dendritic spines. Our observations support the idea of a sequential loss of dendritic spines in association with the intracellular tau pathology, occurring first in the distal and then in the more proximal regions. For example, we observed that in neurons showing an intermediate state of neurofibrillar pathology (pattern IIa) there is not necessarily a loss of dendritic spines but a reduction in the density of dendritic spines in the distal segments, commonly accompanied by the accumulation of fibrillar phospho-tauAT8. Finally, neurons with pattern IIb (with well developed intraneuronal neurofibrillary tangles) had a relatively small dendritic tree, with thin dendrites and very few or no dendritic spines, which may simply be due to a more advanced stage of atrophy (**Figures 7** and **8**). Thus, assuming the widely accepted hypothesis that neuronal degeneration and atrophy are progressive, our observations suggest that such processes begin with synaptic disconnection of the dendritic regions distal to the soma.

This progressive disconnection has important functional implications since there is a spatial segregation of synaptic connections. This is well established for the proximal, intermediate and distal dendrites of pyramidal neurons in the hippocampus, where neurons in all CA fields establish different types of synaptic contacts in each of the strata that they traverse (Andersen et al., 2007), but it may also occur in other cortical regions [see Merino-Serrais et al. (2013) and references contained therein]. We proposed that the functional consequences of the cellulipetal disconnection are likely to be selective and progressive in the cerebral cortex as a whole, at least at the cellular level. Finally, we also observed that there are some dendritic branches within a given neuron that are more affected than others, indicating that this cellulipetal disconnection does not occur synchronously across the whole dendritic arbor (see **Figures 7A–C**).

Summarizing, the fact that neuronal loss in EC layer II seems to occur earlier in the course of the disease and to a greater degree than that in layer III, together with the fact that the granule cells of the DG, CA3 thorny excrescences and the apical and basal dendrites of hippocampal CA1 pyramidal neurons are among the earliest targets of this pathology, it is reasonable to hypothesize that the ECII → dentate gyrus → CA3 → CA1 pathway is more susceptible to a premature degeneration. Certainly, ECIII axons form synapses on the most distal portion of the apical dendrites that end in the *stratum lacunosum-moleculare*, which also appears to be affected in the early stages of the disease. However, the remaining portions of the apical dendrites and the basal dendrites are heavily innervated by CA3 pyramidal cells (**Figure 6**) and by pyramidal cell axons from CA2, which in turn receives ECII input. Thus, the pathway originating in layer II of the EC seems to be the main candidate for the early onset of AD. However, further studies are necessary to determine whether alterations of granule cells; the degeneration of apical and basal dendrites of CA1 pyramidal neurons; and EC layer II neuronal loss are causal events or independent consequences of the disease occurring in parallel.

## Conflict of Interest Statement

The authors declare that the research was conducted in the absence of any commercial or financial relationships that could be construed as a potential conflict of interest.
